# Prevalence and Factors Associated with GLP-1 Receptor Agonist Use for Weight Management Among Overweight and Obese Adults in the Eastern Province of Saudi Arabia

**DOI:** 10.3390/healthcare14030345

**Published:** 2026-01-29

**Authors:** Khalid Alhussain, Zainab Alshakhs, Layla Albaqshi, Fawatim Alshaqaqiq, Mohammed Alrabiah, Rina Tripathi

**Affiliations:** 1 Department of Pharmacy Practice, College of Clinical Pharmacy, King Faisal University, Al-Ahsa 31982, Saudi Arabia; zzalshakhs@gmail.com (Z.A.); fawatem1312@gmail.com (F.A.); rtripathi@kfu.edu.sa (R.T.); 2 Department of Outpatient Pharmacy, Almoosa Rehabilitation Hospital, Al-Ahsa 31982, Saudi Arabia; laylaj116@gmail.com; 3 Al-Dawaa Pharmacy, Al-Ahsa 31982, Saudi Arabia; phmaar1446@gmail.com

**Keywords:** GLP-1 RAs, obesity, overweight, weight management, weight loss

## Abstract

**Objectives**: This study aimed to assess the prevalence and patterns of GLP-1 receptor agonist (GLP-1 RA) use for weight loss and to identify factors associated with their use among overweight and obese adults in the Eastern Province of Saudi Arabia. **Methods**: A cross-sectional study was conducted among overweight and obese adults aged 18 years and older residing in the Eastern Province. Data were collected in December 2024 using an online, self-administered questionnaire. Descriptive statistics were used to assess the prevalence of GLP-1 RA use, while chi-square tests and logistic regression analyses were performed to examine associations between GLP-1 RA use and relevant independent variables. **Results**: A total of 1264 participants were included. The lifetime prevalence of GLP-1 RA use was 18.2%, with 14.2% reporting current use. Injectable semaglutide (Ozempic) was the most commonly used medication (73.9%). Most individuals reported initiating treatment based on a doctor’s recommendation (70.4%), although 36.5% began use based on personal decision. Multivariable logistic regression identified several factors significantly associated with GLP-1 RA use, including obesity status, diabetes, prior weight-loss attempts, having family members or friends who use GLP-1 RAs, and studying or working in a health-related field. **Conclusions**: GLP-1 RA use is substantial among overweight and obese adults in the Eastern Province. In addition to medical conditions, social influences and involvement in health-related fields appear to shape use. These findings emphasize the need for awareness initiatives promoting appropriate GLP-1 RA use and stricter adherence to prescription guidelines.

## 1. Introduction

Despite the substantial public health efforts to reduce overweight and obesity in Saudi Arabia, these conditions remain highly prevalent. In 2024, 42.6% of individuals aged 15 years and older were classified as overweight, and 23.1% were classified as obese [1]. These individuals are at significantly increased risk of chronic noncommunicable diseases, particularly cardiovascular disease and type 2 diabetes [2,3,4,5,6,7]. To mitigate this health problem and its associated consequences, multiple approaches exist for weight management including lifestyle modification, pharmacological therapy, and bariatric surgery [8]. Among these, pharmacological interventions, particularly glucagon-like peptide-1 receptor agonists (GLP-1 RAs), have gained increasing attention due to their demonstrated clinical efficacy. A recent systematic review and meta-analysis reported that GLP-1 RA treatment resulted in a mean weight reduction of 2.69 kg and a BMI reduction of 1.22 kg/m^2^ compared with placebo among overweight or obese individuals with type 2 diabetes. Even greater effects were observed in individuals with overweight or obesity without diabetes, with a mean weight reduction of 9.19 kg and a BMI reduction of 2.96 kg/m^2^ [9].

Considering their demonstrated efficacy, several GLP-1 RAs, including liraglutide, dulaglutide, and semaglutide, are used for weight management worldwide. For example, approximately 3% of adults in Great Britain reported using a GLP-1 RA for weight loss within the past year, with substantial interest among those considering future use [10]. Among the GLP-1 RAs, semaglutide has been found to be the most commonly used agent for weight management locally and globally [11,12,13,14,15,16,17,18]. Semaglutide is available in two formulations: oral (Rybelsus) and injectable (Ozempic); both were approved by the US Food and Drug Administration (FDA) for type 2 diabetes management [19]. Although Ozempic is indicated for type 2 diabetes, it has been widely used off-label for weight loss, even in patients without diabetes. In 2021, the US FDA approved semaglutide specifically for weight management under the brand name Wegovy, which was later registered in Saudi Arabia in January 2025 [19,20]. In addition to the traditional GLP-1 RAs, tirzepatide, a GLP-1 RA with additional glucose-dependent insulinotropic-peptide (GIP) activity, was approved by the US FDA as a therapeutic option for weight management in 2022. Tirzepatide has demonstrated a greater efficacy in weight reduction compared to traditional GLP-1 RAs, establishing it as a promising next-generation medication for obesity management [21].

Given the growing role and clinical significance of the GLP-1 RAs, assessing their utilization in Saudi Arabia is essential. Previous studies conducted in Saudi Arabia have primarily examined awareness, perceptions, and knowledge of GLP-1 RAs [22,23], with only a few reporting their use in Jeddah and Tabuk [12,13]. Jeddah and Tabuk are important cities in Saudi Arabia; however, their demographic profiles differ from that of the Eastern Province, which may influence patterns of weight-loss medication use. The Eastern Province has the largest population among the three regions, with a relatively young population and a marked male predominance, reflecting its large industrial and energy-sector workforce. Jeddah, a major metropolitan and commercial center, has an older population and the highest proportion of non-Saudis, consistent with its diverse, service-oriented labor market. Tabuk has a smaller population, a younger age profile, a higher proportion of Saudi citizens, and a more balanced sex distribution [24]. In terms of healthcare infrastructure, specifically university hospitals, these three regions vary and remain less extensive than the capital, Riyadh, which hosts the most comprehensive network of university hospitals. Jeddah contains the largest academic medical center in western Saudi Arabia, King Abdulaziz University Hospital, with over 1000 beds and numerous specialized clinics [25]. The Eastern Province is served by King Fahd Hospital of the University, a major referral center [26]. Tabuk is developing its own university hospital under the University of Tabuk to enhance medical education and regional healthcare capacity [27]. These differences in population size, age structure, sex distribution, nationality composition, and healthcare infrastructure indicate notable regional variation in socioeconomic characteristics, healthcare access, and health-seeking behaviors, highlighting the need for region-specific data from the Eastern Province. Thus, examining GLP-1 RA use for weight management in the Eastern Province, where data remain limited, is needed. Moreover, no study has investigated factors associated with GLP-1 RA use among overweight and obese adults in Saudi Arabia. Understanding these factors is crucial for informing clinical practice and public health strategies. Therefore, the objectives of this study were to assess the prevalence and patterns of GLP-1 RA use for weight loss and to identify factors associated with their use among overweight and obese adults in the Eastern Province of Saudi Arabia.

## 2. Materials and Methods

### 2.1. Study Design and Participants

A cross-sectional study was conducted through an anonymous, online, self-administered questionnaire. The study was performed in accordance with the principles of the Research Ethics Committee at King Faisal University, which granted its ethical approval to the study protocol (KFU-REC-2024-DEC-ETHICS2914). Eligible participants were adults aged 18 years or older residing in the Eastern Province of Saudi Arabia, who were able to read and write in Arabic and classified as either overweight (BMI 25–29.9), or obese (BMI ≥ 30). Individuals who did not meet these criteria or who declined to participate were excluded from the study. Additionally, extreme outliers in BMI values, defined as those beyond three interquartile ranges from the first or third quartile, were excluded from the analysis.

### 2.2. Questionnaire Development

The questionnaire was developed based on the study objectives and refined through pilot testing with a diverse sample of 15 participants, varying in age, gender, and educational background. Participants were asked to provide feedback on the clarity of the survey to ensure it was understandable to the general public. Feedback collected during this process was used to revise and improve the questionnaire prior to distribution. The final questionnaire consisted of three main sections: (1) demographics and health information; (2) GLP-1 RA use; and (3) factors related to weight management.

The first section included questions on demographics (e.g., age, gender, marital status), socioeconomic status (e.g., income, health insurance), and health information such as height and weight. The second section focused on the use of weight loss medications. Participants who indicated they had used such medications were asked to provide detailed information, including whether their use was current or in the past, the names of the medications, the year they began using them, and their body weight prior to starting the medications. Current users were asked: “Which of the following medications are you currently using to lose weight?” while past users were asked: “Which of the following weight loss medications have you used before?” Thus, current use was defined as active use at the time of survey completion, while past use referred to prior use that had been discontinued. Response options included: Ozempic (semaglutide), Mounjaro (tirzepatide), Saxenda (liraglutide), Rybelsus (semaglutide), and “Other.” Participants who reported current or past use were also asked about the source of their decision to use these medications, including recommendations from doctors, family or friends, social media, or self-initiated use. Participants could select more than one source of recommendation. They were also asked whether medication costs were paid out of pocket or covered by insurance. In the third section, participants reported whether they had tried other weight loss methods, including dieting, exercise, or surgery. Participants were also asked if they knew friends or family members who were currently using or had previously used weight loss medications (e.g., Ozempic, Mounjaro, Saxenda, Rybelsus). The final survey was distributed via commonly used social media platforms in Saudi Arabia, including WhatsApp and X (formerly Twitter), between 7 and 31 December 2024. The survey link was shared through public posts and group messages to maximize reach among residents of the Eastern Province.

### 2.3. Statistical Analysis

Descriptive statistics were used to assess the prevalence of GLP-1 RA use among study participants. Chi-square tests were used to examine the bivariate associations between GLP-1 RA use and the independent variables. Logistic regression models were performed to identify factors associated with GLP-1 RA use. All prespecified covariates were included simultaneously using the Enter method. Multicollinearity was assessed using variance inflation factors (VIFs), and no evidence of problematic multicollinearity was observed (all VIFs < 5). For predictors with low event counts, standard logistic regression estimates may be unstable. To evaluate the robustness of these estimates, bootstrap logistic regression was performed, generating 2000 resampled datasets and computing bias-corrected and accelerated (BCa) confidence intervals for the regression coefficients. Standard logistic regression odds ratios are reported in the main text, while bootstrap results are provided in the Supplementary Materials. All analyses were conducted using IBM SPSS Statistics (version 25).

## 3. Results

### 3.1. Characteristics of the Study Sample

**Figure 1:**
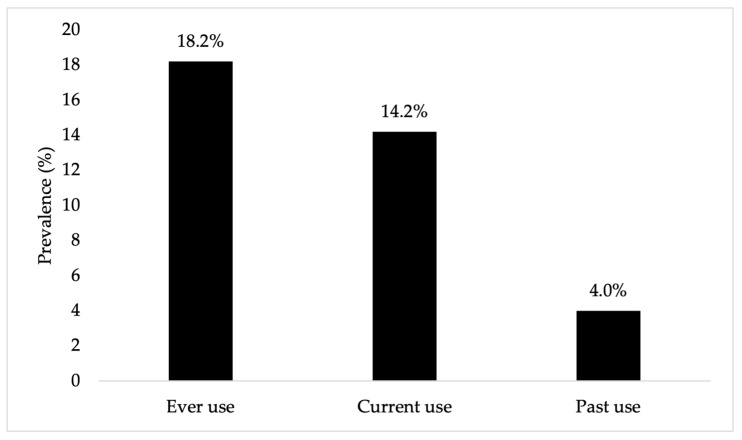
Prevalence of GLP-1 RA Use Among Overweight and Obese Adults in the Eastern Region of Saudi Arabia (n = 1264).

The study sample consisted of 1264 overweight and obese adults residing in the Eastern Province of Saudi Arabia. Based on BMI classification, 55.5% were overweight and 44.5% were obese, yet only 14.7% reported having been diagnosed with obesity by a doctor. The majority of participants (64.3%) resided in Al Ahsa, 52.7% were men, and 44.9% were aged 18–34 years. In our sample, 36.7% were studying or working in health-related fields, and 69.7% were employed. In terms of comorbidities, 23.0% reported a diagnosis of diabetes. About 38.4% reported knowing family members or friends who had used GLP-1 RAs. All sample characteristics are presented in Table 1. The lifetime prevalence of GLP-1 RA use for weight loss was 18.2%, with 14.2% reporting current use and 4.0% reporting past use (see Figure 1).

### 3.2. GLP-1 RA Users

Data on GLP-1 RA users (n = 230) are provided in Table 2. Ozempic was the most used medication, reported by 73.9%, followed by Mounjaro (20.0%), and Rybelsus (7.0%). Among the users, participants could select more than one source for their decision to use GLP-1 RAs. Accordingly, 70.4% reported using GLP-1 RAs based on a doctor’s recommendation, 36.5% based on self-decision, and 15.2% based on family members/friends’ recommendations. Regarding the payment method, 59.6% were covered by insurance or governmental support, while 40.4% paid out-of-pocket.

### 3.3. Factors Associated with GLP-1 RA Use

Bivariate chi-square tests revealed statistically significant associations between GLP-1 RA use and other variables (see Table 3). In terms of sociodemographic variables, married participants reported higher GLP-1 RA use (20.9%) than unmarried participants (11.6%) (*p* < 0.001). Participants classified as obese according to BMI had a significantly higher percentage of GLP-1 RA use compared to those classified as overweight (26.1% vs. 11.8%, *p* < 0.001). Among participants diagnosed with obesity, 42.5% reported ever using GLP-1 RAs versus 14.0% of those without an obesity diagnosis (*p* < 0.001). In terms of comorbidities, 37.5% of participants with diabetes reported ever using GLP-1 RAs, while 12.4% of those without diabetes did (*p* < 0.001). We also found that a significantly greater proportion of participants with dyslipidemia (35.5%) reported GLP-1 RA use compared to those without dyslipidemia (16.6%) (*p* < 0.001). Regarding weight loss efforts, participants who reported attempting dieting had a higher percentage of GLP-1 RA use than those who did not (22.4% vs. 12.9%, *p* < 0.001). Likewise, participants who reported attempting a surgical procedure for weight loss had a significantly higher percentage of GLP-1 RA use than those who did not (48.8% vs. 17.2%, *p* < 0.001). Moreover, we observed that participants who knew family members or friends using GLP-1 RAs reported a higher percentage of use (31.1%) compared to their counterparts (10.2%) (*p* < 0.001).

In the multivariable logistic regression, several variables were found to be independently associated with GLP-1 RA use (see Table 4). Regarding obesity-related variables, participants classified as obese according to BMI were 2.06 times as likely to use GLP-1 RAs as those classified as overweight (95% CI = 1.40, 3.02). In addition, participants diagnosed with obesity were 3.63 times as likely to use GLP-1 RAs as those without an obesity diagnosis (95% CI = 2.34, 5.62). Participants diagnosed with diabetes were also more likely to use GLP-1 RAs compared to those without diabetes (AOR = 5.02; 95% CI = 3.34, 7.54). Regarding weight loss efforts, participants who reported attempting a surgical procedure for weight loss had significantly higher odds of using GLP-1 RAs than those who did not (AOR = 2.26, 95% CI = 1.03, 5.00). Similarly, participants who reported attempting dieting were more likely to use GLP-1 RAs than those who did not (AOR = 2.11, 95% CI = 1.37, 3.24). In contrast, participants who reported attempting to lose weight through exercise were 41% less likely to use GLP-1 RAs than their counterparts (AOR = 0.59; 95% CI = 0.38–0.90). We observed that participants who knew family members or friends using GLP-1 RAs were 3.46 times as likely to use GLP-1 RAs compared to those who did not (AOR = 3.46, 95% CI = 2.38, 5.03). Participants studying or working in health-related fields were 1.67 times as likely to use GLP-1 RAs compared to those in non-health-related fields (95% CI = 1.12, 2.49). Geographically, participants residing in Dammam were 76% less likely to use GLP-1 RAs compared to those from other eastern cities, excluding Al Ahsa and Qatif (AOR = 0.24, 95% CI = 0.12, 0.47). Likewise, Al Ahsa residents were 43% less likely to use GLP-1 RAs compared to those from other eastern cities, excluding Dammam and Qatif (AOR = 0.57, 95% CI = 0.33, 0.98).

Because prior surgical weight-loss attempts, heart disease, and anxiety/depression were rare predictors, we assessed the stability of their estimates using bootstrap analysis. The association between prior surgery and GLP-1 RA use remained positive. However, the confidence interval was wide (AOR = 2.26, 95% CI = 0.75, 6.35), reflecting uncertainty due to the small number of participants with prior surgery. For heart disease, bootstrap estimates were less precise, with confidence intervals including 1 (AOR = 0.67, 95% CI = 0.30, 1.31). Similarly, for anxiety/depression, bootstrap estimates were less precise (AOR = 1.38, 95% CI = 0.66, 2.81), highlighting the need for caution in interpreting these results. Full bootstrap results are provided in Supplementary Table S1.

## 4. Discussion

The present study investigated the prevalence and patterns of GLP-1 RAs as well as factors associated with their use for weight management among overweight and obese adults in the Eastern Province of Saudi Arabia. The lifetime prevalence of GLP-1 RA use in our overweight and obese sample was 18.2%, with 14.2% reporting current use. These estimates are lower than the 21.8% prevalence reported in a recent survey study in Tabuk City; however, that study included both obese and non-obese individuals, which may contribute to higher overall prevalence [13]. The study conducted in Jeddah described GLP-1 RA use among adults with elevated BMI [12]. Because it included only individuals already using these medications and did not estimate prevalence in the general population, its findings are not directly comparable with our results. Compared with other countries, GLP-1 RA use for weight management appears higher in Saudi Arabia. For example, a study from Great Britain reported an overall prevalence of 4.5%, with 2.9% of participants using GLP-1 RAs to support weight loss and 1.7% using them exclusively for this purpose [10]. This could be due to stricter prescription regulations in Western countries, where these medications are less accessible without a valid prescription. It may also be influenced by healthcare providers’ preference for GLP-1 RAs as first-line agents in the treatment of obesity in Saudi Arabia [14]. These findings provide important insight into the emerging landscape of GLP-1 RA use and its potential future demand. Use of these agents has risen across the Kingdom, mirroring trends in other high-income countries [15], driven by the high national prevalence of obesity and diabetes [28]. Rapid adoption may also be supported by increased drug availability, growing clinician and public awareness, strong patient demand, and supportive health policies.

Among GLP-1 RAs, injectable semaglutide (Ozempic) was the most frequently used medication in our sample, consistent with previous studies from both local and international studies [10,13,14,15,16,17]. This pattern may reflect both the well-documented efficacy of semaglutide in promoting weight loss and the fact that Wegovy had not been registered in Saudi Arabia at the time of data collection; it was only approved by the Saudi FDA in January 2025 [20,29,30,31]. Medication preferences can vary internationally due to differences in regulatory approval, clinical guidelines, insurance coverage, market availability, and clinician familiarity. For example, tirzepatide has recently emerged as the leading therapy for weight management in Great Britain [10]. This pattern may evolve over time as international research continues to advance GLP-1 RA therapies through the development of novel agents, combination incretin treatments, and improved formulations aimed at enhancing efficacy, tolerability, and adherence [32,33]. While these therapies are increasingly optimized and utilized for obesity management, their long-term benefits and safety should be evaluated and compared with alternative treatment strategies. A recent meta-analysis of five cohort studies comparing bariatric surgery with GLP-1 RA therapy in adults with obesity found that surgery was associated with lower risks of all-cause mortality, major adverse cardiovascular events, and heart failure compared with GLP-1 RAs [34], emphasizing the need for randomized trials to provide definitive comparative evidence. Ongoing international research and development may further influence the future adoption of GLP-1 RAs in Saudi Arabia.

Regarding the demographic characteristics, GLP-1 RA use was highest among individuals aged ≥ 35 years and lowest among young adults. This association was observed in the bivariate analysis but was not present after adjusting for other factors. This finding may be explained by the national data indicating that the mean age of patients with diabetes in Saudi Arabia ranges from 53 to 59 years [35,36], while the mean age of individuals with obesity is approximately 36.4 years [37]. Similarly, higher GLP-1 RA use among females compared with males was observed in the bivariate analysis. However, this association was not statistically significant after adjustment, indicating that sex alone does not independently predict GLP-1 RA uptake.

In terms of the source of recommendation for GLP-1 RA use, most users in our sample initiated treatment based on their doctor’s advice. However, a notable proportion reported using GLP-1 RAs following self-decision, recommendations from family or friends, or influence from social media. This pattern raises concerns regarding potential off-label use without appropriate medical supervision. Unsupervised use of GLP-1 RAs may increase the risk of inappropriate dosing, unrecognized contraindications, and adverse drug events. GLP-1 RAs are classified as prescription medications by the Saudi FDA [38]; nevertheless, they may still be obtained without a prescription through community pharmacies or other sources. In response to this issue, the Saudi Ministry of Health launched a regulatory campaign in August 2025 to monitor and enforce compliance with prescription requirements for weight-loss medications, enhance patient safety, and reduce health risks associated with unsupervised use. The Ministry has highlighted serious health risks associated with inappropriate use, such as pancreatitis and long-term gastrointestinal disorders, and has urged individuals to consult specialists before using these medications. Healthcare providers and pharmacies have been reminded to adhere strictly to regulatory requirements, with violations subject to fines or license revocation. The public is also encouraged to report non-compliance to help protect public health [39]. Ongoing enforcement will be important to sustain compliance, and the impact of the regulatory campaign should be systematically evaluated to determine its effectiveness in reducing inappropriate GLP-1 RA use and improving patient safety. Efforts to strengthen enforcement should be complemented by educational campaigns that balance caution about unsupervised use with encouragement of evidence-based, medically supervised therapy for obesity and diabetes. Such initiatives could inform the public about the clinical benefits and risks of GLP-1 RAs and promote appropriate medical supervision. They could also address misinformation from informal sources and ensure that individuals who may benefit from these treatments receive accurate guidance and care. With respect to affordability and access, we found that over half of the GLP-1 RA users were covered by insurance, whereas approximately 40% paid out of pocket, consistent with reports from other regional studies [40]. Given the high cost of GLP-1 RAs, out-of-pocket payment may represent a substantial financial burden and could adversely affect treatment initiation, adherence, and continuation. Financial considerations may have discouraged use among non-users or contributed to discontinuation among past users. Variability in insurance coverage and access to GLP-1 RAs in the Kingdom is likely influenced by factors such as employment sector, citizenship status, and the limited reimbursement policies for high-cost medications, which may exacerbate disparities in access.

Several factors were found to be independently associated with GLP-1 RA use. Consistent with prior research, obesity, defined by BMI or physician diagnosis, and diabetes were significant predictors [12,13,17,41]. It is worth noting that BMI-based obesity was substantially higher than physician-diagnosed obesity, which could be due to underdiagnosis, reliance on clinical judgment, patient perception of weight, and variability in documentation. This discrepancy highlights the need for more systematic obesity screening in clinical practice. Obesity and diabetes are key drivers of GLP-1 RA use, as physicians often prioritize this therapy in diabetic patients who are overweight or obese due to its dual efficacy in glycemic control and weight reduction. This clinical rationale likely explains the positive association observed in our study. Furthermore, prior weight-loss attempts, particularly bariatric surgery and dieting, were positively associated with GLP-1 RA use, whereas patients who reported relying on exercise alone were less likely to use these agents. These findings highlight how previous weight-loss experiences influence both patients’ readiness and physicians’ decisions to initiate GLP-1 RAs, with those having a history of surgical or dietary interventions more likely to adopt pharmacological approaches for sustained weight management [12,29]. The lower likelihood of GLP-1 RA use among individuals who reported attempting exercise for weight loss may be explained by the Health Belief Model [42]. These individuals may perceive lower susceptibility to obesity-related health risks or view exercise as an adequate strategy. They may also perceive greater barriers to GLP-1 RA therapy including cost, injections, or side effects. These perceptions may reduce motivation to initiate GLP-1 RAs. Moreover, we found that social and professional factors may influence GLP-1 RA use. Knowing a family member or friend using GLP-1 RAs and involvement in a health-related field, either through work or study, were each independently associated with use. These associations may reflect greater awareness of and confidence in the effectiveness of these medications. Individuals often adopt treatments when observing benefits among peers, and those in health-related fields may be more proactive in seeking care and may have earlier access through professional networks. Within the Eastern Province, variations in GLP-1 RA use were observed, likely reflecting differences in healthcare accessibility, physician prescribing patterns, and patient awareness.

This study has both strengths and limitations. To our knowledge, it is the first study to examine factors associated with GLP-1 RA use for weight management among overweight and obese adults in Saudi Arabia. Another strength was the inclusion of a broad range of demographics, clinical, social, and behavioral factors in the multivariable analysis, which allowed for a better understanding of use patterns. However, several limitations should be acknowledged. The cross-sectional design limits the ability to infer causal relationships. All data were self-reported and may be subject to recall and social desirability biases. The use of an online convenience sampling approach may introduce sampling bias, thereby limiting the representativeness of the sample. Compared with the Eastern Province population, younger adults aged 18–34 years were overrepresented (44.9% vs. 38.0%), and married individuals comprised a higher proportion of the sample (71.3% vs. 57.4%), while men were underrepresented (52.7% vs. 63.8%) [24]. These differences suggest that prevalence estimates may be skewed toward individuals with internet access and a particular interest in weight-loss medications. Moreover, the region-specific sampling approach may further restrict the generalizability of the findings to populations in other regions. Finally, the survey did not collect detailed clinical information such as treatment duration, dosing, adherence, or side effects since these were beyond the scope of the current analysis. While such data are important for evaluating treatment quality and clinical outcomes, future longitudinal studies incorporating detailed clinical information are needed to better evaluate the safety and effectiveness of GLP-1 RA therapy in real-world settings.

## 5. Conclusions

This study provides critical insights into the local patterns and factors influencing GLP-1 RA use for weight management among overweight and obese adults in the Eastern Province of Saudi Arabia. The observed prevalence of GLP-1 RA use aligns with rising global and national trends. Our findings also highlight the significant influence of factors such as obesity status, diabetes comorbidity, prior weight-loss attempts, knowing someone who uses GLP-1 RAs, and engagement in health-related fields on medication utilization. These results emphasize the need to enhance awareness of appropriate GLP-1 RA use to support informed and effective treatment decisions, as well as the necessity for stricter enforcement of dispensing regulations.

## Figures and Tables

**Table 1:** Characteristics of the Study Sample (n = 1264).

Variables	n (%)
Age	
18–34 years	568 (44.9%)
35–54 years	546 (43.2%)
55 years and above	150 (11.9%)
Gender	
Male	666 (52.7%)
Female	598 (47.3%)
BMI classification	
Overweight	701 (55.5%)
Obese	563 (44.5%)
Area of residence	
Al Ahsa	813 (64.3%)
Dammam	254 (20.1%)
Qatif	78 (6.2%)
Other	119 (9.4%)
Educational level	
Less than bachelor	730 (57.8%)
Bachelor	405 (32%)
Greater than bachelor	129 (10.2%)
Marital status	
Married	901 (71.3%)
Unmarried	363 (28.7%)
Income	
None	147 (11.6%)
5000 SR or less	312 (24.7%)
5001–12,000 SR	279 (22.1%)
More than 12,000 SR	223 (17.6%)
Prefer not to say	303 (24%)
Health insurance	
Yes	738 (58.4%)
No/I don’t know	526 (41.6%)
Employment status	
Employed	881 (69.7%)
Unemployed	383 (30.3%)
Studying or working in health-related fields	
Yes	464 (36.7%)
No	800 (63.3%)
Comorbidities	
Diabetes Mellitus	291 (23%)
Diagnosed obesity	186 (14.7%)
Dyslipidemia	110 (8.7%)
Anxiety/depression	79 (6.3%)
Heart disease	80 (6.3%)
Dieting attempt for weight loss	
Yes	705 (55.8%)
No	559 (44.2%)
Exercising attempt for weight loss	
Yes	750 (59.3%)
No	514 (40.7%)
Surgical attempt for weight loss	
Yes	41 (3.2%)
No	1223 (96.8%)
Knowing family members/friends using GLP-1 RAs	
Yes	486 (38.4%)
No/I don’t know	778 (61.6%)

Note: Other cities included Abqaiq, Dhahran, Khobar, Saihat, Safwa, Jubail, Nairyah, Ras Tanura, and Hafr Al Batin. Abbreviations: BMI: Body Mass Index; GLP-1 RAs: glucagon-like peptide-1 receptor agonists.

**Table 2:** Characteristics and Use Patterns Among GLP-1 RA Users (n = 230).

Variables	n (%)
Type of GLP-1 RA used	
Ozempic (Injectable Semaglutide)	170 (73.9%)
Mounjaro (Tirzepatide)	46 (20.0%)
Rybelsus (Oral Semaglutide)	16 (7.0%)
Saxenda (Liraglutide)	14 (6.1%)
Source of recommendation for GLP-1 RA use	
Doctor	162 (70.4%)
Self-decision	84 (36.5%)
Family members or friends	35 (15.2%)
Social media	12 (5.2%)
Payment method for GLP-1 RAs	
Insurance/governmental support	137 (59.6%)
Out-of-pocket	93 (40.4%)

Note: Percentages may not total 100% as respondents were allowed to select multiple options. Abbreviations: GLP-1 RAs: glucagon-like peptide-1 receptor agonists.

**Table 3:** Characteristics of the Study Sample by GLP-1 RA Use Status in Saudi Arabia, 2024 (n = 1264).

	GLP-1 RA Users (n = 230)	Non-GLP-1 RA Users (n = 1034)	*p*-Value
Age			0.007
18–34 years	82 (14.4%)	486 (85.6%)	
35–54 years	114 (20.9%)	432 (79.1%)	
55 years and above	34 (22.7%)	116 (77.3%)	
Gender			0.018
Male	105 (15.8%)	561 (84.2%)	
Female	125 (20.9%)	473 (79.1%)	
BMI classification			<0.001
Overweight	83 (11.8%)	618 (88.2%)	
Obese	147 (26.1%)	416 (73.9%)	
Area of residence			0.001
Al Ahsa	142 (17.5%)	671 (82.5%)	
Dammam	34 (13.4%)	220 (86.6%)	
Qatif	18 (23.1%)	60 (76.9%)	
Other	36 (30.3%)	83 (69.7%)	
Educational level			0.016
Less than bachelor	121 (16.6%)	609 (83.4%)	
Bachelor	74 (18.3%)	331 (81.7%)	
Greater than bachelor	35 (27.1%)	94 (72.9%)	
Marital status			<0.001
Married	188 (20.9%)	713 (79.1%)	
Unmarried	42 (11.6%)	321 (88.4%)	
Income			0.001
None	24 (16.3%)	123 (83.7%)	
5000 SR or less	34 (10.9%)	278 (89.1%)	
5001–12,000 SR	51 (18.3%)	228 (81.7%)	
More than 12,000 SR	52 (23.3%)	171 (76.7%)	
Prefer not to say	69 (22.8%)	234 (77.2%)	
Health insurance			0.043
Yes	148 (20.1%)	590 (79.9%)	
No/I don’t know	82 (15.6%)	444 (84.4%)	
Employment status			0.013
Employed	176 (20%)	705 (80%)	
Unemployed	54 (14.1%)	329 (85.9%)	
Studying or working in health-related fields			0.002
Yes	105 (22.6%)	359 (77.4%)	
No	125 (15.6%)	675 (84.4%)	
Diabetes Mellitus			<0.001
Yes	109 (37.5%)	182 (62.5%)	
No	121 (12.4%)	852 (87.6%)	
Diagnosed obesity			<0.001
Yes	79 (42.5%)	107 (57.5%)	
No	151 (14%)	927 (86%)	
Dyslipidemia			<0.001
Yes	39 (35.5%)	71 (64.5%)	
No	191 (16.6%)	963 (83.4%)	
Anxiety/depression			0.046
Yes	21 (26.6%)	58 (73.4%)	
No	209 (17.6%)	976 (82.4%)	
Heart disease			0.464
Yes	17 (21.3%)	63 (78.8%)	
No	213 (18%)	971 (82%)	
Dieting attempt for weight loss			<0.001
Yes	158 (22.4%)	547 (77.6%)	
No	72 (12.9%)	487 (87.1%)	
Exercising attempt for weight loss			0.002
Yes	116 (15.5%)	634 (84.5%)	
No	114 (22.2%)	400 (77.8%)	
Surgical attempt for weight loss			<0.001
Yes	20 (48.8%)	21 (51.2%)	
No	210 (17.2%)	1013 (82.8%)	
Knowing family members/friends using GLP-1 RAs			<0.001
Yes	151 (31.1%)	335 (68.9%)	
No/I don’t know	79 (10.2%)	699 (89.8%)	

Notes: The *p*-values were obtained from Chi-square tests. Abbreviations: BMI: Body Mass Index; GLP-1 RAs: glucagon-like peptide-1 receptor agonists.

**Table 4:** Adjusted Odds Ratios and 95% Confidence Intervals of Study Sample Characteristics from Logistic Regressions on GLP-1 RA Use.

	AOR	95% CI	*p*-Value
Age			
18–34 years	0.96	[0.50, 1.85]	0.910
35–54 years	0.94	[0.52, 1.68]	0.825
55 years and above (Ref.)			
Gender			
Male	0.80	[0.54, 1.17]	0.249
Female (Ref.)			
BMI classification			
Obese	2.06	[1.40, 3.02]	<0.001
Overweight (Ref.)			
Area of residence			
Al Ahsa	0.57	[0.33, 0.98]	0.044
Dammam	0.24	[0.12, 0.47]	<0.001
Qatif	0.99	[0.44, 2.25]	0.989
Other (Ref.)			
Educational level			
Less than bachelor	0.92	[0.51, 1.67]	0.777
Bachelor	0.89	[0.50, 1.60]	0.703
Greater than bachelor (Ref.)			
Marital status			
Married	1.34	[0.83, 2.17]	0.233
Unmarried (Ref.)			
Income			
5000 SR or less	0.65	[0.31, 1.35]	0.252
5001–12,000 SR	0.91	[0.41, 1.99]	0.808
More than 12,000 SR	0.97	[0.42, 2.24]	0.937
Prefer not to say	0.99	[0.46, 2.15]	0.984
None (Ref.)			
Health insurance			
Yes	0.92	[0.62, 1.36]	0.677
No/I don’t know (Ref.)			
Employment status			
Employed	1.58	[0.92, 2.73]	0.097
Unemployed (Ref.)			
Studying or working in health-related fields			
Yes	1.67	[1.12, 2.49]	0.012
No (Ref.)			
Diabetes Mellitus			
Yes	5.02	[3.34, 7.54]	<0.001
No (Ref.)			
Diagnosed Obesity			
Yes	3.63	[2.34, 5.62]	<0.001
No (Ref.)			
Dyslipidemia			
Yes	1.09	[0.63, 1.87]	0.764
No (Ref.)			
Anxiety/depression			
Yes	1.38	[0.73, 2.63]	0.324
No (Ref.)			
Heart disease			
Yes	0.67	[0.33, 1.36]	0.271
No (Ref.)			
Dieting attempt for weight loss			
Yes	2.11	[1.37, 3.24]	0.001
No (Ref.)			
Exercising attempt for weight loss			
Yes	0.59	[0.38, 0.90]	0.015
No (Ref.)			
Surgical attempt for weight loss			
Yes	2.26	[1.03, 5.00]	0.043
No (Ref.)			
Knowing family members/friends using GLP-1 RAs			
Yes	3.46	[2.38, 5.03]	<0.001
No/I don’t know (Ref.)			

Notes: Based on 1264 overweight and obese adults aged 18 years or older residing in the Eastern Region of Saudi Arabia. Abbreviations: AOR: adjusted odds ratio; CI: confidence interval; Ref.: reference group; BMI: Body Mass Index; GLP-1 RAs: glucagon-like peptide-1 receptor agonists.

## Data Availability

The data presented in this study are available upon request from the corresponding author. The data are not publicly available due to privacy and ethical restrictions related to human participant data.

## Supplementary Materials

The following supporting information can be downloaded at: https://www.mdpi.com/article/10.3390/healthcare14030345/s1, Table S1. Bootstrap-Adjusted Odds Ratios (AORs) and Bias-Corrected and Accelerated (BCa) 95% Confidence Intervals from Multivariable Logistic Regression Examining Factors Associated with GLP-1 RA Use.
