# Supplementary material for: Prevalence and Factors Associated with GLP-1 Receptor Agonist Use for Weight Management Among Overweight and Obese Adults in the Eastern Province of Saudi Arabia
Source: Healthcare (Basel). 2026 Jan 29;14(3):345. doi: 10.3390/healthcare14030345 (PMC12897281; doi:10.3390/healthcare14030345)
Supplement: Supplementary file 1 [file healthcare-14-00345-s001.zip › healthcare-4076658-supplementary.pdf]

Supplementary Table S1. Bootstrap-Adjusted Odds Ratios (AORs) and Bias-Corrected and Accelerated (BCa) 95% Confidence Intervals from Multivariable Logistic Regression Examining Factors Associated with GLP-1 RA Use.

	AOR	BCa 95% CI	P-value
**Age**			
18-34 years	0.96	[0.44, 2.18]	0.922
35-54 years	0.94	[0.50, 1.77]	0.828
55 years and above (Ref.)			
**Gender**			
Male	0.80	[0.53, 1.15]	0.249
Female (Ref.)			
**BMI classification**			
Obese	2.06	[1.40, 3.02]	<0.001
Overweight (Ref.)			
**Area of residence**			
Al Ahsa	0.57	[0.31, 1.03]	0.059
Dammam	0.24	[0.12, 0.42]	<0.001
Qatif	0.99	[0.41, 2.30]	0.990
Other (Ref.)			
**Educational level**			
Less than bachelor	0.92	[0.49, 1.86]	0.774
Bachelor	0.89	[0.48, 1.73]	0.703
Greater than bachelor (Ref.)			
**Marital status**			
Married	1.34	[0.82, 2.40]	0.265
Unmarried (Ref.)			
**Income**			
5000 SR or less	0.65	[0.29, 1.36]	0.282
5001-12000 SR	0.91	[0.38, 2.10]	0.833
More than 12000 SR	0.97	[0.40, 2.31]	0.933
Prefer not to say	0.99	[0.43, 2.44]	0.985
None (Ref.)			
**Health insurance**			
Yes	0.92	[0.60, 1.39]	0.700
No/I don't know (Ref.)			
**Employment status**			
Employed	1.58	[0.87, 3.24]	0.138
Unemployed (Ref.)			
**Studying or working in health-related fields**			
Yes	1.67	[1.03, 2.86]	0.018
No (Ref.)			
**Diabetes Mellitus**			
Yes	5.02	[2.98, 9.29]	<0.001
No (Ref.)			
**Diagnosed Obesity**			
Yes	3.63	[2.14, 6.68]	<0.001
No (Ref.)			
**Dyslipidemia**			
Yes	1.09	[0.56, 2.16]	0.776
No (Ref.)			
**Anxiety/depression**			
Yes	1.38	[0.68, 2.68]	0.296

No (Ref.)			
**Heart disease**			
Yes	0.67	[0.28, 1.41]	0.292
No (Ref.)			
**Dieting attempt for weight loss**			
Yes	2.11	[1.26, 3.77]	0.001
No (Ref.)			
**Exercising attempt for weight loss**			
Yes	0.59	[0.36, 0.87]	0.032
No (Ref.)			
**Surgery attempt for weight loss**			
Yes	2.26	[0.81, 6.28]	0.073
No (Ref.)			
**Knowing family members/friends using GLP-1 RAs**			
Yes	3.46	[2.25, 5.96]	<0.001
No/I don't know (Ref.)			

Notes: Bootstrap results are based on 2000 bootstrap samples.
Abbreviations: AOR: adjusted odds ratio; CI: confidence interval; Ref.: reference group; BMI: Body Mass Index; GLP-1: glucagon-like peptide-1 receptor agonists.
